# A Randomized, Double-Blind Placebo Controlled Trial of Balapiravir, a Polymerase Inhibitor, in Adult Dengue Patients

**DOI:** 10.1093/infdis/jis470

**Published:** 2012-07-17

**Authors:** Nguyet Minh Nguyen, Chau Nguyen Bich Tran, Lam Khanh Phung, Kien Thi Hue Duong, Huy le Anh Huynh, Jeremy Farrar, Quyen Than Ha Nguyen, Hien Tinh Tran, Chau Van Vinh Nguyen, Laura Merson, Long Truong Hoang, Martin L. Hibberd, Pauline P. K. Aw, Andreas Wilm, Niranjan Nagarajan, Dung Thi Nguyen, Mai Phuong Pham, Truong Thanh Nguyen, Hassan Javanbakht, Klaus Klumpp, Janet Hammond, Rosemary Petric, Marcel Wolbers, Chinh Tran Nguyen, Cameron P. Simmons

**Affiliations:** 1 Oxford University Clinical Research Unit; 2 Hospital for Tropical Diseases, Ho Chi Minh City, Vietnam; 3 Centre for Tropical Medicine, Nuffield Department of Medicine, University of Oxford, Oxford, United Kingdom; 4 Genome Institute of Singapore, Singapore; 5 Hoffmann-La Roche, Nutley, New Jersey

**Keywords:** Dengue, therapeutics, clinical trial, anti-viral

## Abstract

***Background.*** Dengue is the most common arboviral infection of humans. There are currently no specific treatments for dengue. Balapiravir is a prodrug of a nucleoside analogue (called R1479) and an inhibitor of hepatitis C virus replication in vivo.

***Methods.*** We conducted in vitro experiments to determine the potency of balapiravir against dengue viruses and then an exploratory, dose-escalating, randomized placebo-controlled trial in adult male patients with dengue with <48 hours of fever.

***Results.*** The clinical and laboratory adverse event profile in patients receiving balapiravir at doses of 1500 mg (*n* = 10) or 3000 mg (*n* = 22) orally for 5 days was similar to that of patients receiving placebo (*n* = 32), indicating balapiravir was well tolerated. However, twice daily assessment of viremia and daily assessment of NS1 antigenemia indicated balapiravir did not measurably alter the kinetics of these virological markers, nor did it reduce the fever clearance time. The kinetics of plasma cytokine concentrations and the whole blood transcriptional profile were also not attenuated by balapiravir treatment.

***Conclusions.*** Although this trial, the first of its kind in dengue, does not support balapiravir as a candidate drug, it does establish a framework for antiviral treatment trials in dengue and provides the field with a clinically evaluated benchmark molecule.

***Clinical Trials Registration.*** NCT01096576.

Dengue is an acute illness caused by 1 of 4 single-stranded positive-sense RNA viruses and is the commonest arboviral infection of humans. In countries where dengue is endemic, the case burden strains already fragile healthcare systems and has an economic cost [1, 2]. There are currently no licensed vaccines for dengue (although late-stage trials are in progress), and mosquito vector control has been mostly unsuccessful or unsustainable.

Clinically apparent dengue manifests with a spectrum of symptoms. High fever, erythema, headache, and myalgia are common symptoms, and laboratory findings of leukopenia and mild thrombocytopenia are typical. The critical phase occurs around the time of defervescence, typically on days 4–6 of illness, during which a transient capillary permeability syndrome manifests in some patients. In children particularly, capillary permeability can be significant enough to precipitate life-threatening circulatory shock, called dengue shock syndrome (DSS). Treatment is supportive, and the mortality rate for DSS in experienced hospital settings is <1% [2].

The magnitude of the early dengue virus (DENV) burden in patients with dengue has been associated with overall clinical outcome. For example, the early plasma viremia and/or NS1 antigenemia levels in pediatric dengue patients who develop clinically significant capillary permeability are higher than in patients without this complication [3–6]. The higher antigenic burden in these patients is believed to trigger a cascade of immunological events that promotes capillary permeability [7]. The association between high viral burdens in the first few days of illness and more severe outcomes has encouraged antiviral discovery efforts for dengue [8, 9], with the rationale that a reduction of the viral burden should result in a reduced incidence of severe complications and a lessening of symptoms and illness duration.

Balapiravir is a prodrug of a nucleoside analogue (4′-azidocytidine) called R1479 and was developed for the treatment of chronic hepatitis C Virus (HCV) infection by Hoffmann-La Roche. [10–12]. Monotherapy twice per day for 14 days reduced plasma HCV levels in a dose- and time-dependent manner and was well-tolerated at doses up to 3000 mg in adult male patients [13]. However, the clinical development of balapiravir for HCV infection was stopped when clinical safety signals were detected in patients receiving extended courses (2–3 months) of balapiravir therapy in conjunction with pegylated interferon and ribavirin. Because HCV and DENV possess RNA-dependent RNA polymerases that share a similar overall architecture [14], we explored a new indication for balapiravir by testing the in vitro activity of R1479 against DENV. Subsequently, the safety, tolerability, and antiviral efficacy of balapirivir in adult dengue patients were investigated in a clinical trial.

## METHODS

### Ethics Statement

The study was conducted according to International Conference on Harmonisation Good Clinical Practice guidelines. All patients provided written informed consent. The trial protocol was approved by the Oxford University Tropical Research Ethical Committee and the Scientific and Ethical Committee of the Ministry of Health, Vietnam. The trial was registered at http://www.clinicaltrials.gov (NCT01096576).

### Clinical Studies

#### Patient Enrollment

Adult men attending the outpatient department of the Hospital for Tropical Diseases, Ho Chi Minh City, Vietnam, who presented with clinical suspicion of dengue confirmed by positive NS1 rapid test (NS1 STRIP, Bio-Rad) were invited to participate in the study. The patient inclusion criteria for study enrollment were (1) male patient aged 18–65 years, (2) history or presence of fever (temperature, ≥38°C) coinciding with a clinical suspicion of DENV infection and a positive NS1 rapid test, (3) onset of symptoms <48 hours prior to initial dosing, (4) and body mass index of 18–35. Two forms of contraception were required for male patients and their partners of childbearing potential, and written informed consent was obtained before any study-specific procedures were performed.

The patient exclusion criteria included (1) clinically significant abnormal laboratory test results which were deemed to be unassociated with dengue infection, or alternatively were diagnostic of dengue shock syndrome; (2) clinical evidence or a history of clinically significant respiratory, metabolic, cardiac, renal, hepatic, gastrointestinal, hematological, neurological, psychiatric, or chronic disease; (3) positive test result for human immunodeficiency virus (HIV) at screening; (4) history of autoimmune or immune dysfunction disease; (5) neutrophil count of <1500 cells/mm^3^, Haemoglobin (Hgb) concentration of <13 g/dL, or platelet count of <90 000 cells/mm^3^ at screening; (6) calculated creatinine clearance of <80 mL/minutes; (7) hypertension or hypotension; (8) pulse pressure of <20 mm Hg; (9) essential concomitant medication with the exception of paracetamol; (10) positive test result for drugs of abuse or alcohol; (11) recent participation in an investigational drug or device study; and (12) not being a suitable candidate for enrollment in the opinion of the investigator or sponsor.

#### Randomization, Masking, Dosing Schedule, and Dose Escalation Cohorts

Randomization to treatment group was by computer-generated randomization sequencing in blocks of 2. Balapiravir was formulated in a 500-mg film-coated tablet. The placebo consisted of an identical looking tablet containing excipient only. All balapiravir and placebo tablets were provided by F. Hoffmann-La Roche. Patients in cohort 1 (*n* = 20) received 1500 mg of balapiravir or an identical placebo orally every 12 hours for 5 days (10 doses). The decision to dose escalate (to 3000 mg) was made after review of the partially unblinded group mean clinical and laboratory data acquired from patients in cohort 1. Patients in cohort 2 (*n* = 44) received 3000 mg of balapiravir or placebo orally twice a day for 5 days (10 doses).

#### Safety Assessments

Eligible patients remained in clinic during a 7-day in-patient period then returned for follow-up visits on study days 14, 28, and 84. During the inpatient period, patient vital signs were assessed every 6 hours and routine laboratory investigations were performed daily or as clinically indicated. Safety and tolerability were monitored at regular intervals and included physical examinations, vital signs, electrocardiograms, clinical laboratory assessments, incidence of clinical adverse events, and concomitant medications. Any clinically significant abnormal laboratory test results were followed up until resolved or stabilized. A quality of life assessment, using a questionnaire and a visual analog scale, was implemented on study days 1, 3, 5, 7, 14, 28, and 84.

### Clinical Laboratory Investigations

#### Pharmacokinetics

Serum samples for pharmacokinetic investigations were collected at time 0 (predose) and 2, 4, 8, and 12 hours postdose on days 1 and 5. Pharmacokinetics parameters were calculated using noncompartmental methods.

#### Virological and Immunological Measurements

Plasma samples were collected for virological and immunological investigations every 12 hours, beginning immediately before commencement of treatment, during the next 6 study days and on one occasion on study day 7 and day 14 (virological markers) or day 7 and day 28 (immunological markers). Viremia was measured using a validated, internally controlled reverse-transcription polymerase chain reaction assay in a Good Clinical Laboratory Practice environment [15]. The limit of detection was 357 copies/mL for DENV-1, 72 copies/mL for DENV-2, 357 copies/mL for DENV-3, and 720 copies/mL for DENV-4. The presence of NS1 in plasma was determined using the Platelia NS1 assay (Bio-Rad) and was performed according to the manufacturer's instructions. Plasma cytokine levels (interleukin 1β [IL-1β], IL-2, IL-4, IL-5, IL-6, IL-10, IL-12p70, IL-13, interferon γ [IFN-γ], and tumor necrosis factor α [TNF-α]) were measured using a Bio-Plex human cytokine assay (Bio-Rad) and a multiplex array reader (Luminex Systems, Bio-Plex workstation from Bio-Rad Laboratories) according to the manufacturer's instructions. Briefly, 50-μL plasma samples were incubated with monoclonal antibody coupled beads. Complexes were washed twice, then incubated with biotinylated detection antibodies and, finally, labeled with streptavidin-phycoerythrin prior to analysis. Cytokine concentrations in samples were calculated by use of recombinant cytokines as standards and software provided by the manufacturer (Bio-Plex Manager). All research samples (for pharmacology, virology, and immunology) were collected and processed in the laboratory within 1 hour of venupuncture. All virological and immunological measurements and analyses were performed by analysts who were blind to the treatment assignment

### Statistical Methods

All randomized patients in the study were analyzed according to the intention-to-treat principle with 3 treatment groups: 1500 mg of balapiravir, 3000 mg of balapirivir, and placebo (combining the patients in the placebo arms of both cohorts).

Key viremia endpoints of the study were as follows: area under the log-transformed viremia curve (AUC) from first dose to the end of study day 7 (study hour 168), calculated on the basis of the trapezoidal rule with values below the limit of detection replaced by half of the detection limit; time to first viremia level of <1000 copies/mL until study day 7; and time to the first negative NS1 test result. Other predefined key endpoints included fever clearance time, defined as the time from the start of treatment to the start of the first 48-hour period during which axillary temperature remained <37.5°C; maximum hematocrit level; maximum percentage increase of hematocrit level from baseline; platelet count nadir; and lowest recorded quality of life score.

Time to event endpoints were compared between treatment groups on the basis of Cox regression and continuous endpoints on the basis of linear regression. Analyses were adjusted for the predose value of the respective endpoint; viremia and NS1 endpoints were additionally adjusted for dengue serotype. For all endpoints, we report *P* values of a trend test for a dose-response relationship with treatment entered as a continuous variable with values 0 (placebo), 1 (low-dose), and 2 (high-dose) into the regression model.

The sample size of the study was determined by practical and clinical considerations and not based on formal statistical power calculations. However, we used simulation to post hoc estimate the power of this trial to detect different effect sizes, expressed as log_10_ viremia reductions per day due to active drug. We assumed that the effect of high-dose drug is twice as large as the low-dose effect and that the 32 recruited placebo patients are representative of the entire target population. We simulated drug effects of varying size on top of the observed viremia profiles for these 32 patients and then used bootstrap simulation to assess the power of an overall comparison of the AUC of log_10_ viremia adjusted for serotype and baseline log_10_ viremia between study arms (trend test) and of a pairwise comparison of high dose versus placebo. According to these simulations, the study would have had 80% power to detect a true reduction of 0.25 log_10_ viremia per day (ie, one log_10_ reduction over 4 days) in the high-dose group by both the trend test and the pairwise comparison at the 2-sided 5% significance level.

All analyses were performed with the statistical software R, version 2.11.1 (R Foundation for Statistical Computing).

## RESULTS

### In Vitro Inhibition of DENV Replication by R1479 in Huh-7 Cells

R1479, the active nucleoside released from balapiravir, inhibited replication of DENV reference strains and clinical isolates in Huh-7 cells with mean half maximal effective concentration (EC_50_) values of 1.9–11 μM (Supplementary Table 1). Mean values of median inhibitory concentration (IC_50_) for NITD008, a previously characterized nucleoside analogue polymerase inhibitor of DENV [16], were 0.1–0.6 μM (Supplementary Table 1). R1479 was also active against DENV-1, DENV-2, and DENV-4 (DENV-3 was not tested) in primary human macrophages (mean EC_50_ range, 1.3–3.2 μM) and dendritic cells (mean EC_50_ range, 5.2–6.0 μM; data not shown). These data suggest R1479 has activity against DENV in vitro at EC_50_ concentrations pharmacologically attainable in adult humans receiving ≥1500 mg of balapiravir twice per day [13].

### Randomized, Double-Blind Placebo Controlled Trial of Balapiravir in Adult Male Dengue Patients

**Figure 1.**
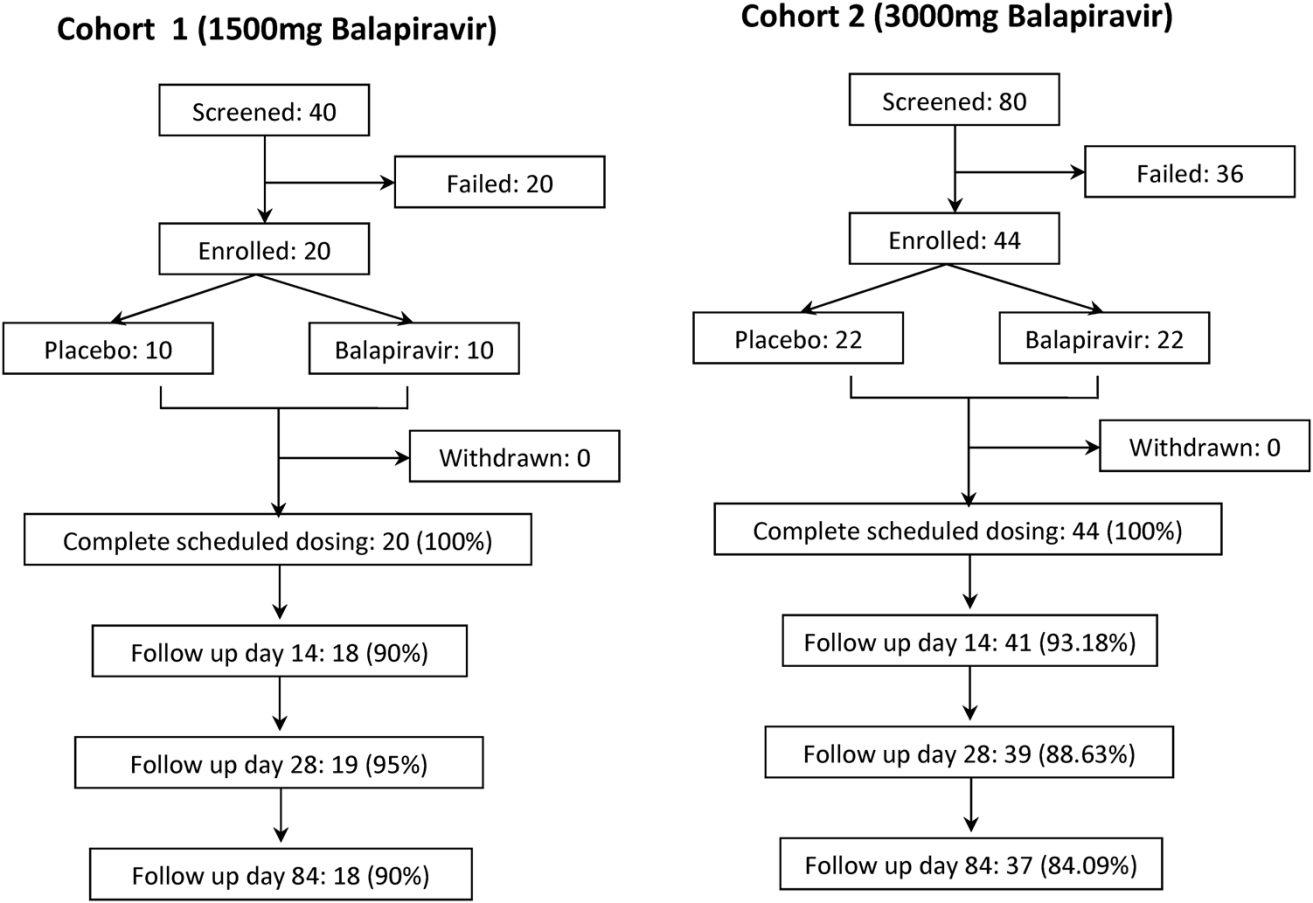
Study enrollment and follow-up. The study enrolled 120 NS1-positive dengue patients who consented and were eligible to undergo screening against the inclusion and exclusion criteria; of these, 64 were eligible to continue into the study. The most common reasons for exclusion were low creatinine clearance rates (53.6% of excluded cases), hepatitis B surface antigen positivity (23.2%), and abnormal serum creatinine level (17.9%). All patients completed their dosage schedule of study drug.

A total of 64 adult patients with dengue were randomly assigned to receive either balapiravir or placebo within 48 hours of symptom onset. Patients in cohort 1 were enrolled from 15 July 2010 through 10 September 2010 and received 1500 mg of balapiravir (*n* = 10) or placebo (*n* = 10) orally twice a day for 5 days (Figure 1). Patients in cohort 2 were enrolled from 1 October 2010 through 16 January 2011 and received 3000 mg of balapiravir (*n* = 22) or placebo (*n* = 22) orally twice a day for 5 days (Figure 1). All enrolled patients completed their schedule of study drug doses.

### Baseline Characteristics of the Patients

The baseline characteristics of the intention-to-treat patient population (*n* = 64 total) were similar in the balapiravir and placebo groups (Table 1). The median duration of illness at the time of commencing treatment was <40 hours for patients in each arm of the study. DENV-1 and DENV-2 accounted for the majority of infections (Table 1). Phylogenetic analyses of genome-length virus sequences determined directly from plasma samples from 40 (62.5%) of 64 patients identified the DENV-1 viruses (*n* = 24 patients) as belonging to the genotype 1 lineage, the DENV-2 viruses (*n* = 11 patients) as belonging to the Asian 1 lineage, and DENV-3 viruses (*n* = 5 patients) as belonging to the genotype 2 lineage (data not shown).

**Table 1.** Baseline Characteristics of the Patient Population

Variable	Placebo (n = 32)	Balapiravir 1500 mg (n = 10)	Balapiravir 3000 mg (n = 22)
Age, years	23 (21.0–28.0)	29.5 (23.5–32.0)	21 (20.0–31.3)
Weight, kg	60 (54.8–65.0)	64.5 (54.6–74.3)	58.5 (53.3–66.4)
Duration of illness at dosing, h	39.25 (36.11–44.89)	33.63 (27.5–45.13)	37.75 (30.85–43.38)
Oral temperature, ^°^C	38.2 (37.6–39.1)	38.8 (38.1–39.3)	38.7 (38.2–39.0)
Serotype, no. of patients/plasma viremia level (range), log_10_ copies/mL			
DENV-1	18/9.07 (4.63–10.52)	5/8.91 (7.50–10.63)	9/9.34 (8.05–9.86)
DENV-2	9/7.89 (6.51–9.58)	4/8.23 (7.57–9.14)	8/8.61 (8.03–9.46)
DENV-3	2/9.79 (9.62–9.96)	0	3/9.07 (8.79–9.17)
DENV-4	3/8.12 (6.12–8.37)	1/5.43 (5.43–5.43)	2/7.84 (7.58–8.10)
Serology, no. (%) of patients			
Primary	4 (12)	1 (10)	0 (0)
Secondary	28 (88)	9 (90)	22 (100)

Data are median (interquartile range), unless otherwise specified.

### Intention-to-Treat Analysis of the Safety and Tolerability of Balapiravir

A summary of the clinically significant adverse events by treatment arm is shown in Supplementary Table 2. No major clinical or laboratory safety signals or significant differences in adverse event profiles between treatment groups were observed. The range of adverse events reported was consistent with the known clinical and laboratory features of dengue. There were 4 reported serious adverse events (SAEs), 2 in the placebo arm and 1 each in the 1500 mg and 3000 mg balapiravir arms (Supplementary Table 2). These SAEs were typical of dengue (2 patients with prolonged thrombocytopenia, 1 patient with transient loss of visual acuity, and 1 patient with narrowed pulse pressure). No SAEs were considered to be dose-related to balapiravir, nor was the dose of study drug altered in response to these SAEs. All SAEs resolved.

### Pharmacological Profile and Antiviral Activity of Balapiravir in Dengue

**Figure 2.**
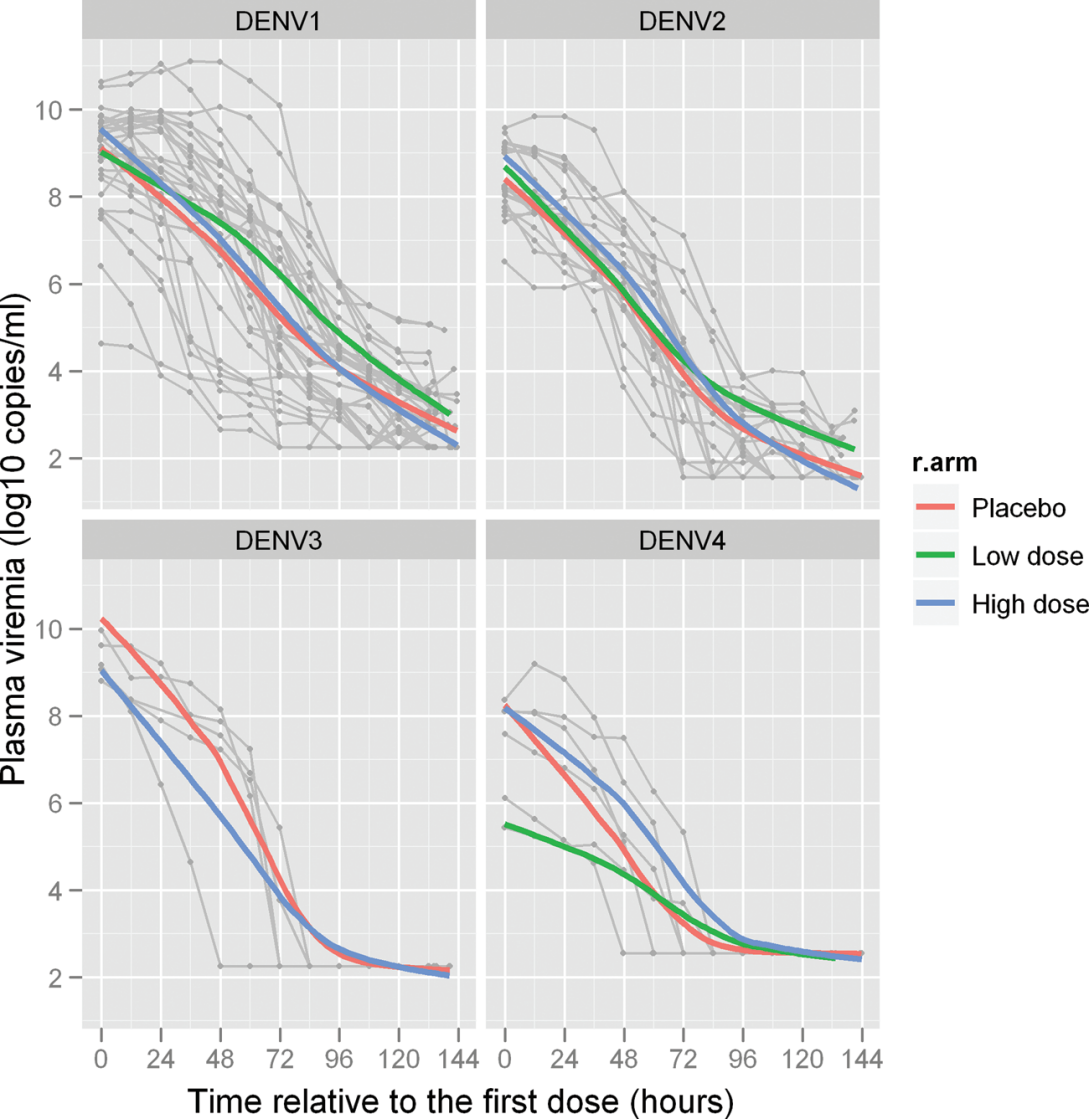
Viremia levels in balapiravir- and placebo-treated patients. Shown are serotype-stratified viremia levels, measured by reverse-transcription polymerase chain reaction, in 12-hour spaced plasma samples, in patients treated with placebo, low-dose balapiravir, or high-dose balapiravir. The colored lines in each graph represent smoothing lines derived from local polynomial regression fitting to data from each treatment arm. The gray background lines represent individual patient data. Abbreviations: DENV, dengue virus; R.arm, randomistation arm.

**Figure 3.**
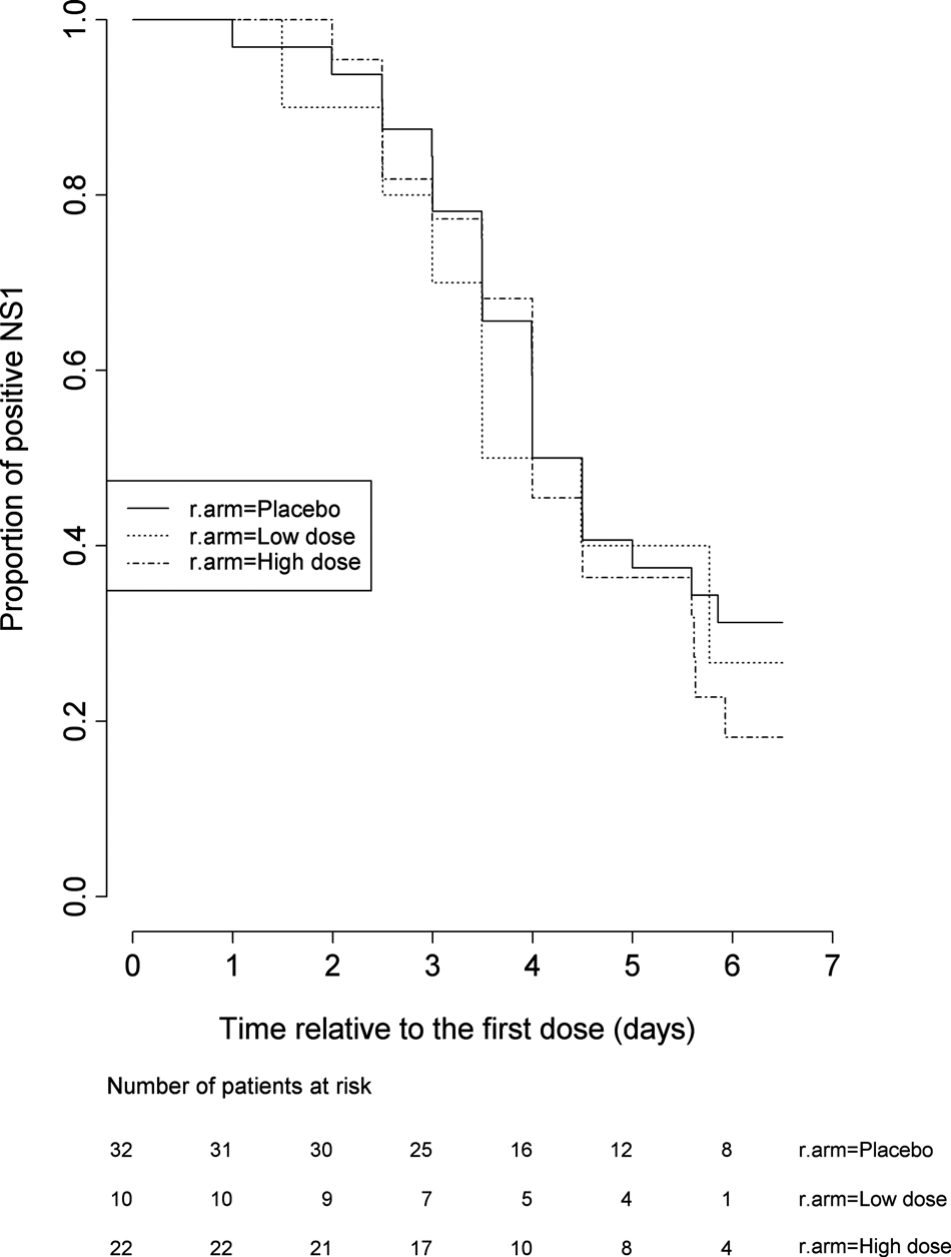
Kaplan-Meier plot of NS1 antigenemia over time in balapiravir- and placebo-treated patients. Shown are the proportions of patients over time that tested NS1-positive in serial plasma samples collected daily from baseline (pretreatment) to study day 7, and again on study day 14. There was no significant difference in time to clearance of NS1 antigenemia between treatment groups. Abbreviation: R.arm, randomistation arm.

In the first 12 hours of treatment, 95% of patients receiving 3000 mg of balapiravir had plasma maximum concentration (*C*_max_) values of R1479 that were >6 μM (Table 2), a concentration that was inhibitory to DENV in vitro (Supplementary Table 1). Values of *C*_max_ were lower in patients receiving 1500 mg of balapiravir, but nonetheless were >6 μM in most patients (Table 2). The C_12 h_ values were 2.7–4.9 μM for 1500 mg and 2.1–15.7 μM for 3000 mg on day 1 of treatment. Similar dose-dependent pharmacological findings were observed on study day 5 (results not shown). Despite the pharmacological evidence that balapiravir treatment elicited dose-dependent levels of R1479 in vivo, there was no measurable effect on the predefined virological endpoints of time to clearance of viremia (Table 3; Figure 2), time to clearance of NS1 antigenemia (Table 3; Figure 3), or AUC (Table 3).

**Table 2.** Pharmacokinetics of R1479 on Study Day 1

Parameter	*T*_max_, h^a^	*C*_max_, μM^b^	*C*_min_, μM^b^	*T*_last_, h^c^	AUC_last_, h × μM
Cohort 1 (1500 mg twice daily)					
Minimum	2	5.46	2.71	12	42.63
Median	4	16.54	3.56	12	110.56
Maximum	8	19.76	4.93	12	130.70
CV, %	49.1	30.5	21.3	…	55.81
Cohort 2 (3000 mg twice daily)					
Minimum	2	8.78	2.14	12	70.05
Median	4	23.86	5.82	12	167.04
Maximum	12	90.60	15.73	12	608.09
CV, %	55.3	60	45.5	…	116.89

Abbreviations: AUC_last_, area under the log-transformed viremia curve; *C*_max_, maximum plasma concentration of R1479 on day 1; *C*_min_, minimum plasma concentration of R1479 on day 1; CV, coefficient of variation *T*_last_, time since treatment when the last sample for pharmacokinetic measurement was collected; *T*_max_, time after treatment when the maximum plasma concentration of R1479 was reached.

**Table 3.** Analysis of Predefined Virological Endpoints in Patients Treated With Balapiravir or Placebo

Endpoint	Placebo (n = 32)	Low-dose Balapiravir (n = 10)	High-dose Balapiravir (n = 22)	*P*
AUC viremia, log_10_ copies/mL × d^a^				.623
Mean	32.78	34.49	32.56	
Median (IQR)	32.19 (26.63–39.24)	29.63 (27.41–39.86)	31.98 (27.60–34.98)	
Median time to first viremia level of <1000 copies/mL, d (IQR)^b^	4 (3–6)	5 (4, NA)	4 (3–5)	.476
Median time to first negative NS1 test result, d (IQR)^b^	4 (3–13)	3 (3–14)	4 (3–6)	.852

^a^ Values of area under the curve (AUC) of log_10_ viremia from day 1 first dose (hour 0) to the end of day 7 (hour 168) calculated with the trapezoidal rule. IQR, interquartile range.

^b^ Kaplan-Meier estimates based on data from the inpatient period only.

### Balapiravir Effects on Clinical Signs and Routine Laboratory Markers

Consistent with a lack of measurable antiviral activity, balapiravir treatment did not affect clinical signs or routine laboratory findings (summary endpoint findings in Table 4). Thus, fever clearance times (Supplementary Figure 1; Table 4) and changes in hematological markers such as platelet count (Table 4) or hematocrit level (Table 4) were not affected by balapiravir treatment. Similarly, the kinetics of biochemical changes (liver transaminase and coagulation marker level) were similar in each of the treatment arms (Table 4). A quality of life score, measured on a visual analogue scale, was also similar between the treatment groups (Table 4). Collectively, these results indicate a lack of measurable effect of balapiravir on these virological, clinical, and routine laboratory markers.

**Table 4.** Analysis of Predefined Clinical and Routine Laboratory Endpoints

Key Endpoint	Placebo (n = 32)	Low-dose Balapiravir (n = 10)	High-dose Balapiravir (n = 22)	*P*
Median fever clearance time, d (IQR)^a^	3 (3–4)	3 (2–4)	3 (2–4)	.286
Maximum recorded hematocrit level, %				
Mean	46.0	46.7	44.6	.102
Median (IQR)	45.9 (44.0–47.3)	45.8 (43.8–49.3)	45.1 (41.8–46.1)	
Maximum percentage increase of hematocrit level, %				
Mean	7.0	11.5	2.6	.131
Median (IQR)	6.7 (0.8–10.0)	11.8 (3.1–15.3)	1.2 (−4.1–9.2)	
Minimum platelet count, 10^9^ cells/L				
Mean	52.6	26.3	54.4	.886
Median (IQR)	47.5 (28.5–64.0)	23 (16.5–33.0)	52 (25.5–64.8)	
Maximum INR				
Mean	1.23	1.17	1.27	.819
Median (IQR)	1.15 (1.08–1.26)	1.16 (1.05–1.26)	1.17 (1.09–1.22)	
Maximum aPTT, s				
Mean	42.2	41.3	41.8	.962
Median (IQR)	40.7 (39.0, 44.5)	40.6 (39.6–43.1)	41.8 (38.3–44.1)	
Minimum fibrinogen level, g/L				
Mean	1.92	1.94	1.88	.902
Median (IQR)	1.99 (1.63–2.14)	2.08 (1.68–2.25)	1.86 (1.60–2.03)	
Maximum AST level, IU/L				
Mean	134.0	143.6	118.4	.646
Median (IQR)	97.0 (70.0–191.5)	157.5 (116.5–171.5)	108.0 (45.0–151.5)	
Maximum ALT level, IU/L				
Mean	111.6	96.3	75.2	.125
Median (IQR)	87.5 (53.3–149.0)	97.5 (58.0–128.8)	68.5 (33.3–104.5)	
Minimum quality of life score^a^				
Mean	76.5	78.3	74.45	.478
Median (IQR)	84.5 (69.8–89.3)	77.0 (69.8–89.0)	77.5 (67.8–82.0)	

Abbreviations: ALT, alanine aminotransferase; aPTT, activated partial thromboplastin time; AST, aspartate aminotransferase; IQR, interquartile range; INR, international normalized ratio.

^a^ Visual analogue scale: 0 is the worst imaginable health state the patient can think of, 100 is the best imaginable health state.

### Balapiravir Effects on the Host Immune Response and Virus Sequence Diversity

Plasma concentrations of TNF-α, IFN-γ, IL-2, IL-4, IL-5, IL-6, IL-10, IL-12p70, IL-13, and IL-1β were measured in serial (daily) plasma samples during the inpatient period and at follow-up (day 28). Concentrations of IFN-γ and IL-10 were transiently elevated during the 48–96 hours after patient enrollment, but this was independent of the treatment assignment. Concentrations of TNF-α, IL-2, IL-4, IL-5, IL-6, IL-12p70, IL-13, and IL-1β were low and did not increase by >2-fold during the treatment period (data not shown). Changes in whole blood gene transcript abundance in serial samples collected at baseline and on study days 4 and 28 were also monitored, but there was no evidence of treatment-related effects on gene transcript abundance in subsequent samples (data not shown). Mutation rates in virus genome sequences in serial plasma samples from patients in the balapiravir and placebo arms were also not significantly different (data not shown). Collectively, these exploratory analyses are consistent with the predefined trial clinical and virological endpoints that suggest balapiravir did not measurably alter the disease evolution of dengue.

## DISCUSSION

The development of antiviral therapies for dengue is, alongside vaccine development and vector control, a rational approach to reducing morbidity and preventing transmission. To this end, here we show that the nucleoside analogue R1479 reduces dengue virus replication in human cells in vitro. Balapiravir, a prodrug of R1479, was also safe and well-tolerated in adult dengue patients who received doses of 1500 mg or 3000 mg twice daily for 5 days. However, balapiravir treatment did not improve virological, immunological, or clinical measures of disease in patients who commenced treatment within the first 48 hours of their illness.

Balapiravir was well tolerated at both 1500 mg and 3000 mg dosage schedules. The range of clinical and laboratory adverse events observed in the balapiravir treatment arms were typical in Vietnamese adults with dengue and not different from those seen in the placebo arm. The rationale for stopping this exploratory study after a total enrollment of 64 patients was that the dengue transmission season was at an end and the number of patients eligible for enrollment had dropped significantly. Second, a planned joint review of the clinical and laboratory data by the sponsor and investigators led to the conclusion that balapiravir was insufficiently potent to warrant further clinical investigation.

Balapiravir treatment in this study was not associated with measurable changes in a range of virological, clinical, or immunological endpoints, despite evidence that the active moiety R1479 had anti-DENV potency in vitro. Several possibilities may explain this. First, the size of the patient cohorts meant that only large effects could have been detected (see the Methods section for further information on effect size). Second, concentrations of R1479 in plasma may have been insufficient to inhibit DENV replication. Pharmacokinetic and pharmacodynamic analyses of balapiravir in hepatitis C showed a good correlation between exposure (measured as AUC_0–12 h_ or *C*_min_) and antiviral effect (measured as reduction in plasma HCV RNA level) [11]. In that study, dosing of 1500, 3000, or 4500 mg of balapiravir twice daily was associated with a dose-dependent increase in R1479 plasma exposure and antiviral effect. A mean plasma trough level 3.5-fold above the human serum adjusted HCV replicon EC_50_ was achieved at the 1500 mg twice daily dose, and this ratio increased to 4.9 and 7.8 in the 2 higher dose groups. In contrast, no significant antiviral effect was apparent at the lower dose of 500 mg twice daily, for which the mean plasma trough level was similar to the HCV replicon EC_50_, even though *C*_max_ was >5-fold above the HCV replicon EC_50_ [11]. With a similar relationship between exposure and antiviral effect in DENV infection, mean *C*_min_ concentrations exceeding 6.7–39 µM or *C*_max_ concentrations exceeding 25–145 µM may be needed for the observation of antiviral effects of R1479 on DENV. This would argue for higher or more frequent doses of balapiravir. Dosing of 4500 mg twice daily of balapiravir achieved mean *C*_min_ of 21 µM and mean *C*_max_ of 88 µM in HCV-infected persons [11]. However, this more aggressive strategy might also be accompanied by increased likelihood of drug-related adverse events as seen previously with short-course monotherapy (4500 mg of balapiravir twice a day in HCV-infected patients) [13]. Third, the antiviral effect of R1479 is dependent on phosphorylation to the DENV polymerase inhibitor R1479 triphosphate. Phosphorylation efficiency can differ between cell types, and R1479 may not be efficiently phosphorylated in the primary DENV target cells in humans. Finally, although the median duration of illness history was <40 hours in all treatment groups, it is possible that the timing of treatment was too late in this patient population to demonstrate a marked antiviral effect above the level that the host adaptive immune response achieves. Indeed, it is striking that decreases in viremia were observed in the majority of patients within 24 hours of enrollment irrespective of the treatment assignment (Figure 2). This underlines the imperative of early diagnosis and treatment with antiviral candidates.

New therapies may require certain properties to be successful for the treatment of an acute infection such as dengue. Antiviral agents must be effective immediately to rapidly control viral replication. Problems of distribution, active metabolite formation, or protein binding may limit the effective response time. Consideration of prophylactic treatment at the first indication of symptoms may be needed to successfully impede viremia, but it will require a compound with a robust safety profile. Although the current study did not furnish evidence that balapiravir at the doses tested is a candidate drug for dengue, it is hoped that this marks the beginning of an intensive period of clinical research on dengue therapeutics.

## Supplementary Data

Supplementary materials are available at *The Journal of Infectious Diseases* online (http://jid.oxfordjournals.org/). Supplementary materials consist of data provided by the author that are published to benefit the reader. The posted materials are not copyedited. The contents of all supplementary data are the sole responsibility of the authors. Questions or messages regarding errors should be addressed to the author.

Supplementary Data
