# Supplementary material for: A Randomized, Double-Blind Placebo Controlled Trial of Balapiravir, a Polymerase Inhibitor, in Adult Dengue Patients
Source: J Infect Dis. 2012 Jul 17;207(9):1442–50. doi: 10.1093/infdis/jis470 (PMC3610419; doi:10.1093/infdis/jis470)
Supplement: Supplementary Data [file supp_207_9_1442__index.html]

A randomized, double-blind placebo controlled trial of balapiravir, a polymerase inhibitor, in adult dengue patients — A Randomized, Double-Blind Placebo Controlled Trial of Balapiravir, a Polymerase Inhibitor, in Adult Dengue Patients — A Randomized, Double-Blind Placebo Controlled Trial of Balapiravir, a Polymerase Inhibitor, in Adult Dengue Patients — Supplementary Data 

# A Randomized, Double-Blind Placebo Controlled Trial of Balapiravir, a Polymerase Inhibitor, in Adult Dengue Patients

## Supplementary Data

Supplementary Data

**Files in this Data Supplement:**

- Supplementary Data - Doc file
