# Supplementary material for: A Randomized, Double-Blind Placebo Controlled Trial of Balapiravir, a Polymerase Inhibitor, in Adult Dengue Patients
Source: J Infect Dis. 2012 Jul 17;207(9):1442–50. doi: 10.1093/infdis/jis470 (PMC3610419; doi:10.1093/infdis/jis470)
Supplement: Supplementary Data [file supp_jis470_jis470supp.doc]

**Supplementary Table 1. Antiviral potency of Balapiravir and 2’E-7D-A (also known as NITD008) against DENV strains in Huh-7 cells.**

|  | **2’E-7D-A** | **Balapiravir** |
| --- | --- | --- |
| **DENV-1 Strain** | **Mean IC50 [μM]^a^** | **Mean IC50 [μM]** |
| **Thsman** | 0.4±0.1 | 8.8±1.2 |
| **FR37^b^** | 0.4±0.1 | 7.2±2.0 |
| **FR42 ^b^** | 0.5±0.2 | 7.4±2.3 |
| **FR28 ^b^** | 0.5±0.1 | 6.8±1.1 |
| **FR34 ^b^** | 0.5±0.2 | 7.0±2.0 |
| **DENV-2 Strain** |  |  |
| **Th36** | 0.5±0.1 | 4.6±0.4 |
| **32/0140 ^b^** | 0.3±0.0 | 1.9±0.7 |
| **32/0102 ^b^** | 0.5±0.2 | 3.0±0.5 |
| **33/0178 ^b^** | 0.3±0.2 | 2.0±0.6 |
| **33/0051 ^b^** | 0.4±0.2 | 2.0±0.2 |
| **DENV-3 Strain** |  |  |
| **H87** | 0.4±0.1 | 3.7±0.9 |
| **33/0072 ^b^** | 0.5±0.2 | 3.0±0.4 |
| **33/0253 ^b^** | 0.6±0.0 | 4.4±0.9 |
| **33/0177 ^b^** | 0.3±0.0 | 3.2±0.9 |
| **33/0096 ^b^** | 0.3±0.1 | 3.4±1.7 |
| **DENV-4 Strain** |  |  |
| **H241** | 0.6±0.1 | 11±1.0 |
| **Phili/1567 ^c^** | 0.1±0.1 | 2.0±0.4 |
| **Venz/JQ ^c^** | 0.2±0.1 | 5.5±1.3 |
| **Senga/DA ^c^** | 0.3±0.1 | 6.5±0.9 |

^a^ Each experiment was done in duplicate. The mean and standard deviation shown represent 3 independent (different date) experiments for each virus tested. Huh-7 cells were plated in 96-well plate at 10,000 cells/well in DMEM containing 10 % FBS in 100 μl volume one day prior to set up of the assay. The following day the medium was aspirated off and diluted virus at a multiplicity of infection of 0.5 or media was added to the assay and control wells, respectively. The virus was allowed to attach to the cells for two hours at 37°C in a humidified 5% C02 atmosphere while the dilutions of the test molecule were prepared. Test molecules (or medium as a control) were added after aspiration of the virus-containing media 2 hours post-infection in 3-fold dilutions at a final DMSO concentration of 1%. After 48 hrs, cells were washed and cellular RNA were isolated. In this study, we used a quantitative real time PCR method to quantitate dengue viral RNA in Huh-7 in the presence of inhibitors. Viral RNA and endogenous 18S rRNA control (Applied Bio System) was quantified by a real time PCR assay using the following primers. Dengue Reverse (Common to all the serotypes): 5’-GATCTCTGGTCTTTCCCAGCGTCAA-3’, Dengue forward serotype 1: 5’-GAGCCCCGTCCAAGGACGTAAAATGAA-3’, Dengue forward serotype 2 or 3: 5’-GAGCCCCGTCCAAGGACGTTAAAAGAA-3’, Dengue forward serotype 4: 5’-TATTGAAGTCAGGCCACTTGTGCC -3’ and Dengue probe (Common to all the serotypes): 5'-/56-FAM/AAGGACTAGAGGTTAGAGGAGACCCCCCGC/3BHQ_1/-3'. Taqman was performed in duplicate. IC_50_ was obtained using the following calculations. First ΔCt was calculated by subtracting 18S rRNA CT value from dengue RNA CT value. ΔCts from duplicate taqman assay were averaged. Then ΔΔCt was obtained by subtracting the average ΔCt of non-treated sample from the treated sample average ΔCt. Relative quantification was calculated using the following formula. Relative quantification = 2^-averageΔΔCT^. The 50% inhibitory concentrations (EC_50_) were calculated using the sigmoidal dose-response model in Microsoft XLfit.

^b^ Low passage clinical isolates from Vietnamese dengue patients

^c^ gift from WRCEVA:Dr. Robert B. Tesh, M.D

**Supplementary Table 2. Summary of adverse events by treatment arm**

no.pt. refers to the number of patients with events.

|  | **Placebo (n=32)** | **Low dose Balapiravir**  **(n=10)** | **High dose Balapiravir (n=22)** |
| --- | --- | --- | --- |
| **ADVERSE EVENT NAME** | **no.pt. (%)** | **no.pt. (%)** | **no.pt. (%)** |
| ANY EVENT   - - no (%) patients with at least one event - - no of events | 25 (78.12%)  94 | 8 (80%)  30 | 19 (86.36%)  65 |
| ABDOMINAL PAIN | 2 (6.25%) | 0 (0%) | 3 (13.64%) |
| ABNORMAL INR | 1 (3.12%) | 0 (0%) | 1 (4.55%) |
| ALTERATION OF TASTE | 1 (3.12%) | 0 (0%) | 0 (0%) |
| ANOREXIA | 2 (6.25%) | 2 (20%) | 1 (4.55%) |
| BRUISING | 3 (9.38%) | 0 (0%) | 2 (9.09%) |
| COUGH | 7 (21.88%) | 0 (0%) | 4 (18.18%) |
| DECREASE OF FIBRINOGEN | 3 (9.38%) | 1 (10%) | 4 (18.18%) |
| DIARRHOEA | 11 (34.38%) | 4 (40%) | 8 (36.36%) |
| DYSPEPSIA | 0 (0%) | 1 (10%) | 1 (4.55%) |
| ELEVATED ALT | 1 (3.12%) | 0 (0%) | 0 (0%) |
| ELEVATED AST | 1 (3.12%) | 0 (0%) | 0 (0%) |
| EPISTAXIS | 2 (6.25%) | 0 (0%) | 0 (0%) |
| FLATULENCE | 1 (3.12%) | 0 (0%) | 0 (0%) |
| GUM BLEEDING | 5 (15.62%) | 0 (0%) | 6 (27.27%) |
| HEADACHE | 5 (15.62%) | 2 (20%) | 5 (22.73%) |
| HEMATEMESIS | 1 (3.12%) | 0 (0%) | 0 (0%) |
| HEMATURIA | 1 (3.12%) | 0 (0%) | 1 (4.55%) |
| HEMOPTYSIS | 1 (3.12%) | 0 (0%) | 0 (0%) |
| HYPOALBUMINEMIA | 1 (3.12%) | 1 (10%) | 1 (4.55%) |
| ITCHY RASH ON CHEEK, AND FOREHEAD | 0 (0%) | 1 (10%) | 0 (0%) |
| NARROW PULSE PRESSURE | 0 (0%) | 1 (10%) ^a^ | 0 (0%) |
| NAUSEA | 3 (9.38%) | 0 (0%) | 2 (9.09%) |
| PETECHIAE | 11 (34.38%) | 4 (40%) | 6 (27.27%) |
| PROLONGED APTT | 6 (18.75%) | 2 (20%) | 7 (31.82%) |
| PROTEINURIA | 1 (3.12%) | 1 (10%) | 0 (0%) |
| PRURITIS | 0 (0%) | 1 (10%) | 0 (0%) |
| RETINAL INJURY | 1 (3.12%) ^a^ | 0 (0%) | 0 (0%) |
| SORE THROAT | 1 (3.12%) | 0 (0%) | 0 (0%) |
| THROMBOCYTOPENIA | 17 (53.12%) ^a^ | 4 (40%) | 11 (50%) ^a^ |
| VERTIGO | 0 (0%) | 1 (10%) | 0 (0%) |
| VOMIT | 5 (15.62%) | 4 (40%) | 2 (9.09%) |

^a^ indicates one patient in this group had a SAE
